# Exogenous Melatonin Boosts Heat Tolerance in *Rosa hybrida* via *RhCOMT1* Modulation

**DOI:** 10.3390/plants14010029

**Published:** 2024-12-25

**Authors:** Chenyang Li, Zhiyin Ding, Zipeng Cai, Yongying Ruan, Peitao Lü, Yang Liu

**Affiliations:** 1 Center for Plant Metabolomics, Haixia Institute of Science and Technology, College of Horticulture, Fujian Agriculture and Forestry University, Fuzhou 350002, China; lichenyang425@163.com; 2 College of Architectural Engineering, Shenzhen Polytechnic University, Shenzhen 518055, China; dingzhiyin@szpt.edu.cn (Z.D.); czp2864054825@163.com (Z.C.); yongyingruan@szpu.edu.cn (Y.R.); 3 National Key Laboratory for Tropical Crop Breeding, Institute of Tropical Bioscience and Biotechnology, Chinese Academy of Tropical Agricultural Sciences, Sanya 572024, China

**Keywords:** *Rosa hybrida*, heat stress, COMT, melatonin, protein translocation

## Abstract

*Rosa hybrida* is one the most commonly cultivated ornamental plant of economic importance and faces major challenges under heat stress. Melatonin has been widely shown to regulate plant stress response; however, the exact mechanism involved in heat stress in *R. hybrida* has yet to be determined. Here, we observed that *R. hybrida* in vitro plantlets supplemented with melatonin in the culture medium exhibited higher chlorophyll content, relative ion leakage, and fresh weight after 12 d of high-temperature treatment; the optimal concentration was established at 5 mg/L. Using molecular and biochemical techniques, we explored the roles of a melatonin synthase gene *RhCOMT1*, which expression was influenced by heat stress and melatonin. RhCOMT1 was located in the nuclear-cytoplasmic under ambient conditions, while heat stress translocated the distribution of RhCOMT1 to chloroplasts. Overexpression of *RhCOMT1* in rose petal enhanced thermotolerance, and silencing of *RhCOMT1* reduced thermotolerance via affect H_2_O_2_ content and relative ion leakage. These findings collectively emphasize the pivotal role of melatonin in enhancing thermotolerance to *R. hybrida* by alleviation of oxidative stress, through modulation of *RhCOMT1* expression and location.

## 1. Introduction

Rose is a world-wide ornamental crop used as cut flowers, garden plants, and potted plants. They are also utilized for industrial purposes such as in perfume production, as well as for medicinal and culinary uses [1]. However, the plant’s sensitivity to high temperature, especially during the flowering stage, poses a significant challenge for the cultivation industry [2]. With the ongoing rise in global temperatures, the consistently high temperatures in summer have become a major environmental factor limiting the growth and development of roses.

Heat stress (HS) is characterized by elevated ambient temperatures surpassing a critical threshold, leading to irreversible harm to plant growth and development [3]. In *R. hybrida*, HS manifests through various effects such as the bent peduncle phenomenon (BPP), leaf discoloration and wilting, flower deformities, disrupted bud differentiation, and other physiological disorders [4]. The adverse impacts of high temperatures stem from the accumulation of reactive oxygen species (ROS), resulting in heightened lipid peroxidation [5]. This process significantly impairs photosynthetic efficiency [6,7] and compromises cell membrane integrity [8], leading to metabolic alterations that influence plant growth and development.

Melatonin functions as a crucial reactive oxygen species (ROS)-scavenging agent [9] and plays a protective role in shielding the photosynthetic machinery from oxidative stress, thereby enhancing plant photosynthetic efficiency [10,11]. Application of exogenous melatonin has enhanced the post-harvest quality of ornamental plants such as carnations [12,13], anthuriums [14], and cut roses [15]. Caffeic acid O-methyltransferase (COMT), belonging to the O-methyltransferase (OMT) family, facilitates the transfer of a methyl group to N-acetylserotonin (NAS), leading to melatonin synthesis [16]. Recent research has demonstrated the involvement of COMT in plant responses to various stresses by modulating melatonin levels, thereby improving yield and quality [17,18,19]. Furthermore, exogenous melatonin application has been shown to boost endogenous melatonin accumulation by upregulating genes related to melatonin synthesis, including COMT, potentially delaying flower senescence [20,21]. However, there is a lack of research investigating the potential of COMT in enhancing the heat stress resilience of ornamental plants, and further exploration of its precise regulatory mechanisms is warranted.

In this study, *R. hybrida* was selected as the experimental material with the aim of providing key evidence that melatonin enhances heat-resistant performance in rose cultivation, as is the regulatory role of *RhCOMT* in the process. In brief, our findings highlighted the importance of melatonin in enhanced thermotolerance through alleviated oxidative stress and imply that it functions via modulation of *RhCOMT1* expression and location.

## 2. Results

### 2.1. Melatonin Enhanced Heat Tolerance in R. hybrida

**Figure 1:**
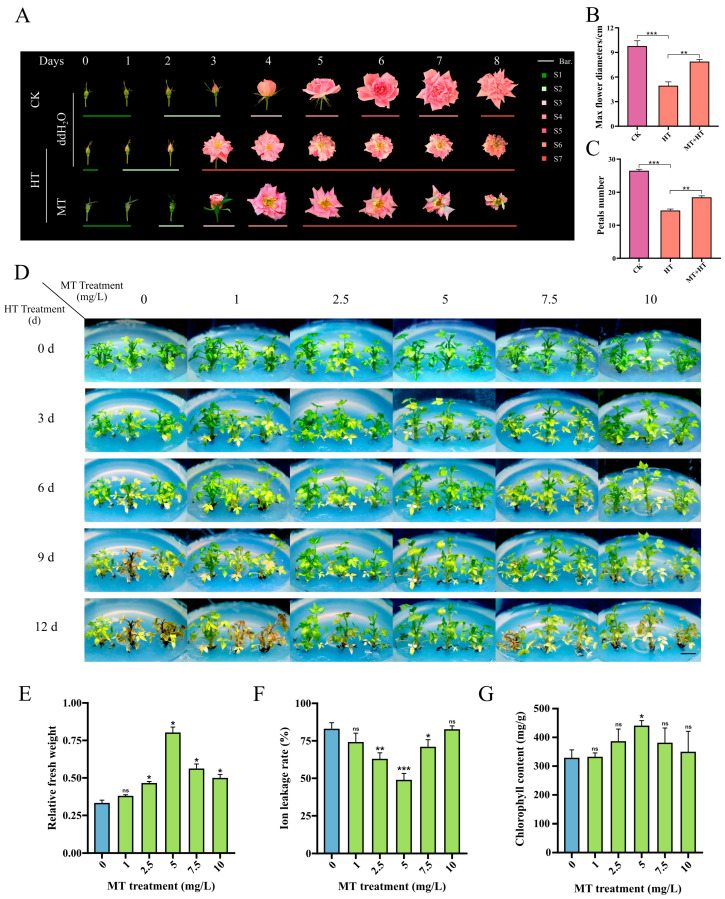
Phenotypic characterization of the buds and sterilized stems under heat stress. (**A**) Phenotypes of the *Rosa hybrida* buds under heat stress. Scale bar = 5 cm. (**B**,**C**) Flower diameters and (**B**) petal numbers (**C**) of CK, HT, MT + HT groups. (**D**) Phenotypes of the *R. hybrida* in vitro sterilized stems in different concentrations of melatonin medium under high-temperature treatment. Scale bar = 1 cm. (**E**–**G**) ChlorophyII content, (**E**) relative ion leakage, and (**F**) relative fresh weight (**G**) of the *R. hybrida* sterilized stems grow in different concentrations of melatonin medium under 12 d of HT. CK, control group; HT, 42 °C high-temperature treatment group; MT, melatonin treatment; error bars represent ± SD (n = 3). Significant differences compared to the 0 mg/L melatonin treatment group are indicated by asterisks (* *p* < 0.05, ** *p* < 0.01, *** *p* < 0.001, ns, no significance, Student’s *t*-test).

To investigate the roles of melatonin in rose petal under heat stress, we chose a temperature-sensitive rose cultivar (*R. hybrida*) under varying temperature conditions and spray treatments. Following the taxonomy proposed by Kuiper et al. (1996) [22], the flowering stages were divided into seven colors. The control group (CK, 25 °C, distilled water) showed a consistent progression in flowering and wilting, while under high-temperature condition (HT, 42 °C, distilled water) exhibiting accelerated desiccation and aging rates, smaller flower diameters, and fewer petal numbers. Notably, following melatonin treatment (HT, 42 °C, melatonin), the control group displayed intermediary characteristics (Figure 1A–C).

We further examined the effects of applying exogenous melatonin to rose plants under heat stress conditions. Under high-temperature treatment at 42 °C, seedlings in the 0 mg/L melatonin group exhibited severe distress, while those treated with melatonin remained viable. The most significant mitigation of high-temperature-induced adverse effects was observed in the group treated with 5 mg/L melatonin (Figure 1D). Analysis of chlorophyll content, relative ion leakage, and relative fresh weight of in vitro seedlings was performed. The presence of melatonin at concentrations of 2.5, 5, 7.5, and 10 mg/L led to a significant increase in the fresh weight of tissue-cultured seedlings exposed to high temperatures (Figure 1E). Additionally, melatonin levels at 2.5, 5, and 7.5 mg/L significantly lowered the ion leakage rate of the seedlings (Figure 1F), with only the application of 5 mg/L of melatonin showing a noteworthy elevation in chlorophyll content in tissue-cultured seedlings subjected to high temperature conditions (Figure 1G). The results indicate that the presence of a 5 mg/L melatonin medium markedly improved the photosynthetic capacity of *R. hybrida* culture seedlings, while also decreasing lipid peroxidation and increasing water content under high-temperature treatment. These results suggest that elevated temperatures severely impair the ornamental value and development of rose plants, but the application of melatonin can mitigate these deleterious effects.

### 2.2. Expression Pattern Analysis and Molecular Characterization of RhCOMT1

**Figure 2:**
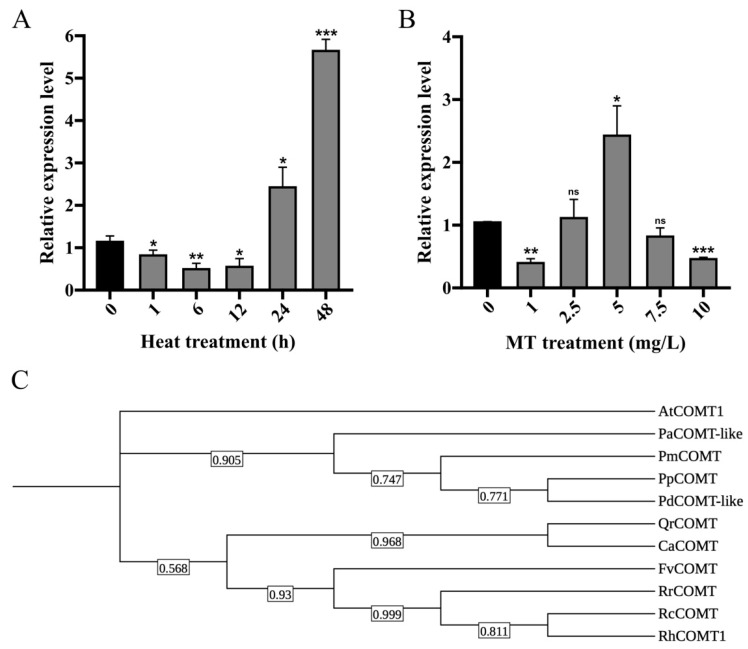
Expression pattern and bioinformatics analysis of *RhCOMT1*. (**A**) The expression levels of *RhCOMT1* under high-temperature treatment. (**B**) The expression levels of *RhCOMT1* under melatonin treatment of different concentrations. (**C**) Phylogenetic relationships between RhCOMT1 and other COMT proteins. The data are presented as the means ± SDs (n = 9). Significant differences compared to the 0 h high-temperature treatment and 0 mg/L melatonin treatment group are indicated by asterisks (* *p* < 0.05, ** *p* < 0.01, *** *p* < 0.001, ns, no significance, Student’s *t*-test).

To delve into the molecular mechanism responsible for the improvement of heat tolerance in roses through melatonin, we analyzed the expression patterns of multiple genes from the rose O-methyltransferase (OMT) family under high-temperature stress (Figure 2 and Figure S1), given the established role of caffeic acid 3-O-methyltransferase in melatonin production [16]. The *RchiOBHmChr7g0187791* transcript showed a decrease in response to heat stress at 12 h, then gradually rising and peaking at 48 h (Figure 2A). Furthermore, the RchiOBHmChr7g0187791 transcript levels were highest at 5 mg/L and lowest at 1 mg/L and 10 mg/L (Figure 2B). These findings suggest that *RchiOBHmChr7g0187791* is responsive to both exogenous melatonin and heat stress. The full-length cDNA of the *RchiOBHmChr7g0187791* gene was cloned, revealing an open reading frame (ORF) of 1062 bp that encodes a peptide of 353 amino acids (XP_024171303.1) with a molecular weight of approximately 38.76 kDa. Phylogenetic analysis showed that *RchiOBHmChr7g0187791* was closely related to *AtCOMT1* in Arabidopsis thaliana; thus, we named it *RhCOMT1* (Figure 2C). Conserved domain blasting showed *RhCOMT1* is highly conserved with COMT proteins in other species, which belonged to the OMT family (Figure S2).

### 2.3. RhCOMT1 Translocalized in Chloroplasts Under Heat Stress

**Figure 3:**
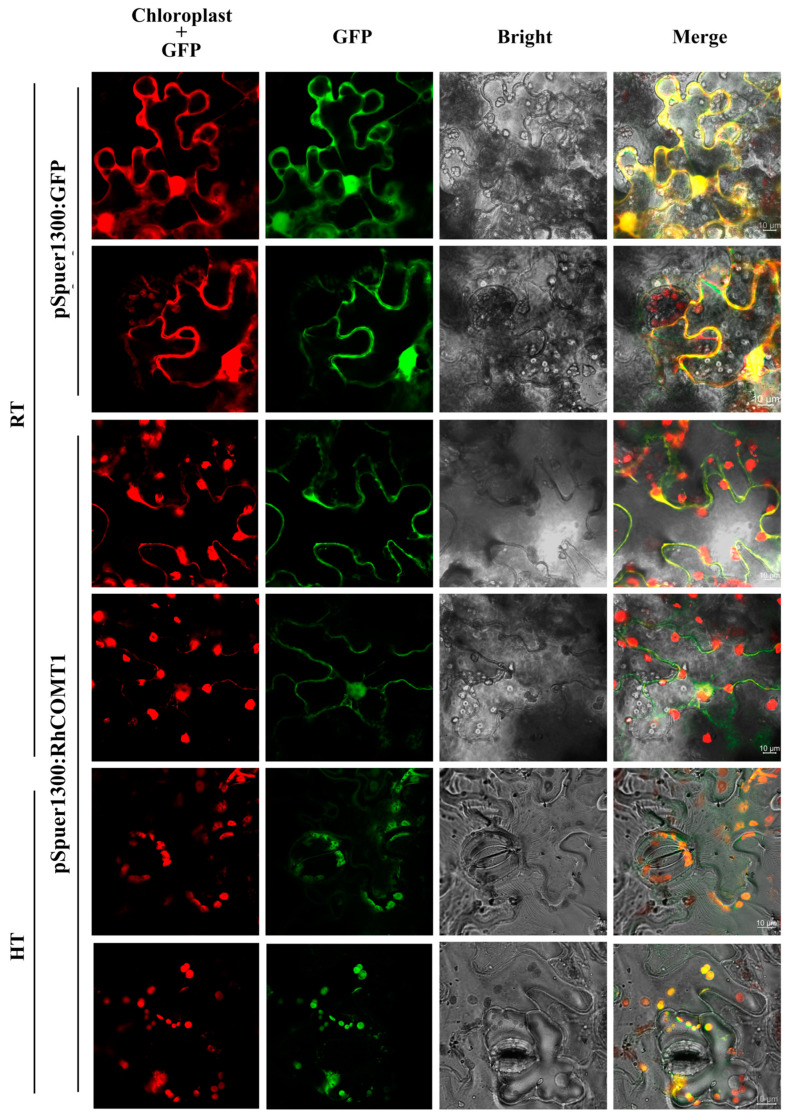
Subcellular localization assay of RhCOMT1 under RT and HT. The location of RhCOMT1 is based on visualization of GFP in tobacco leaves transformed with a fusion construct (pSuper1300:GFP-*RhCOMT1*) or empty vector (pSuper1300:GFP). Microscopic images were taken under bright field and fluorescence. The overlapped images are shown on the right. RT, room temperature; HT, high-temperature treatment; GFP, green fluorescent protein.

The transient expression vector pSuper1300:GFP-*RhCOMT1* was constructed to investigate cellular localization. Separate infiltrations of the fusion plasmids GFP-*RhCOMT1* and control (GFP alone) were conducted in tobacco (*N. benthamiana*) cells, with each treatment comprising three biological replicates (Figure S3). As shown in Figure 3, the GFP-*RhCOMT1* fusion protein was specifically detected in nucleus and cell membrane at RT. However, after high-temperature treatment, the GFP-*RhCOMT1* fusion protein was surprisingly detected in chloroplast. This result indicated that *RhCOMT1* specifically functions in chloroplasts under heat stress.

### 2.4. Overexpression of RhCOMT1 Enhanced R. hybrida Heat Stress Tolerance

**Figure 4:**
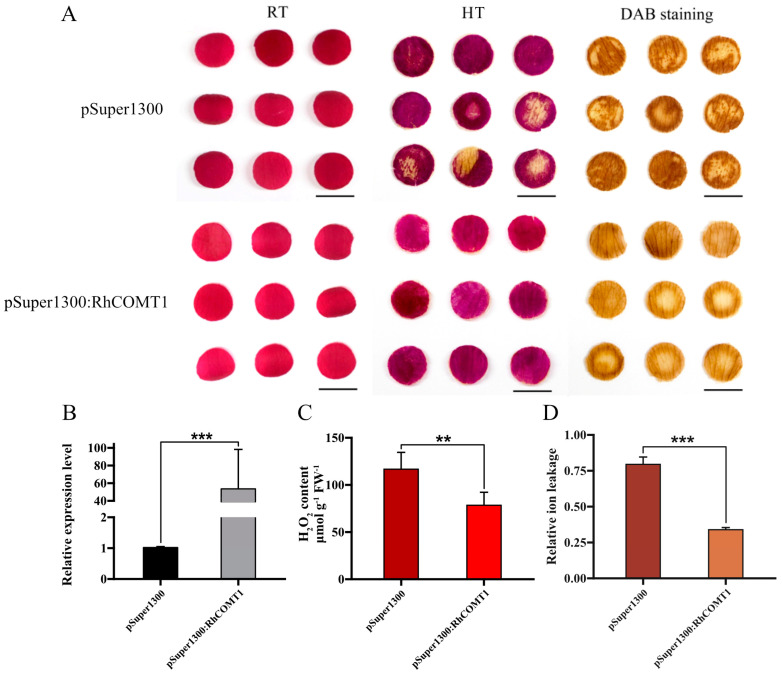
Thermotolerance assay using *RhCOMT1*-overexpressing petal discs. (**A**) The phenotypes of rose petal discs under room temperature (RT, 25 °C) and post high-temperature treatment (HT, 42 °C, 36 h) with DAB staining. The experiment was repeated three times, with 27 rose petal discs per experiment. Scale bar = 1 cm. (**B**) Detection of *RhCOMT1* expression in *RhCOMT1*-overexpression petal discs. (**C**,**D**) Determination of H_2_O_2_ content and (**C**) relative ion leakage (**D**) of petal discs post HT (42 °C, 36 h). The data are presented as the means ± SD for three replicates (** *p* < 0.01, *** *p* < 0.001, Student’s *t*-test, the pSuper1300:*RhCOMT1* compared with the pSuper1300 control post HT, respectively). RT, room temperature; HT, high-temperature treatment; DAB, 3,3′diaminobenzidine.

To assess the biological function of *RhCOMT1* during the heat stress of rose petals, we conducted Agrobacterium-mediated transient transformation to overexpress *RhCOMT1* transcripts in petal discs. Following high-temperature treatment (HT, 42 °C for 36 h), it was observed that rose petals with transient *RhCOMT1* overexpression exhibited significantly less color fading compared to the control group. DAB staining analysis revealed lower levels of reactive oxygen species (ROS) in pSuper1300:*RhCOMT1* plants than in the control plants (Figure 4A). RT-qPCR analysis validated the significant upregulation of the target gene in petals overexpressing *RhCOMT1* (Figure 4B). Moreover, relative ion e leakage and H_2_O_2_ content were notably lower in *RhCOMT1*-overexpressing petals compared to control petals in control group (Figure 4C,D).

### 2.5. Silencing of RhCOMT1 Reduces R. hybrida Tolerance to Heat Stress

**Figure 5:**
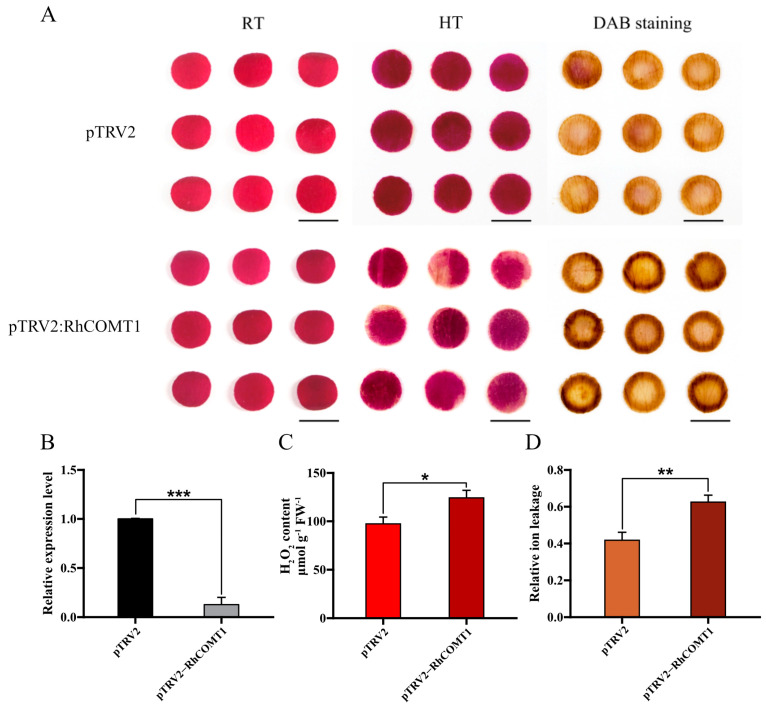
Thermotolerance assay using *RhCOMT1*-silenced petal discs. (**A**) The phenotypes of rose petal discs under room temperature (RT, 25 °C) and high-temperature treatment (HT, 42 °C, 36 h) with DAB staining. The experiment was repeated three times, with 27 rose petal discs per experiment. Scale bar = 1 cm. (**B**) Detection of *RhCOMT1* expression in TRV-VIGS petal discs. (**C**,**D**) Determination of H_2_O_2_ content and (**C**) relative ion leakage (**D**) of petal discs post HT (42 °C, 36 h). The data are presented as the means ± SDs for three replicates (* *p* < 0.05, ** *p* < 0.01, *** *p* < 0.001, Student’s *t*-test, the pTRV2-*RhCOMT1* compared with the pTRV2-control post HT, respectively). RT, room temperature; HT, high-temperature treatment; DAB, 3,3′diaminobenzidine.

In order to further substantiate our findings, we employed TRV (tobacco rattle virus)-VIGS specifically to silence the expression of the *RhCOMT1* in petal discs. The rose petal disc assay revealed that silenced *RhCOMT1* increased color fading after exposure to high temperature (42 °C for 36 h) compared to pTRV2 controls, accompanied by elevated levels of reactive oxygen species (ROS) as revealed by DAB staining (Figure 5A). The expression of *RhCOMT1* was significantly lower in the *RhCOMT1*-silenced discs than in the pTRV2 control discs (Figure 5B). Consistent with this pattern, the silenced discs also presented significantly higher ion leakage and H_2_O_2_ content than did the pTRV2 control discs (Figure 5C,D). These results indicate that *RhCOMT1* functions as a positive regulator of thermotolerance through alleviating oxidative stress and preserving cell membrane integrity in rose.

## 3. Discussion

The disruption of climate patterns and rise in unpredictable high temperatures due to global warming have adverse effects on the growth of *R. hybrida* [23]. Melatonin has emerged as a promising compound for ameliorating heat stress in plants, as highlighted in a comprehensive review by Hassan et al. (2022) [24]. Nevertheless, the precise regulatory mechanism of melatonin in this regard is still not fully understood.

### 3.1. Exogenous Melatonin Enhances Heat Tolerance in R. hybrida Plants and In Vitro Cultures

High-temperature treatment resulted in significant phenotypic changes in *R. hybrida*. However, exogenous melatonin treatment has been demonstrated to mitigate the adverse effects of heat stress on plant health. Exposure to high temperatures led to a notable decrease in physiological well-being (Figure 1A), whereas the group treated with melatonin exhibited a health status more akin to the control group (Figure 1A). In vitro systems offer a valuable platform for studying melatonin receptors and mechanisms in plants due to the ability to closely regulate treatment conditions and maintain cultures under aseptic conditions [25]. Consequently, the phenotypes of *R. hybrida* culture seedlings subjected to different concentrations of melatonin medium under heat stress were examined, revealing that the inclusion of melatonin in the medium significantly extended the survival of tissue culture seedlings under high-temperature conditions, particularly at a concentration of 5 mg/L (Figure 1D). Furthermore, the chlorophyll content, relative conductivity, and plant fresh weight were assessed to evaluate the impact of varying melatonin concentrations on plant heat tolerance (Figure 1E–G). It was observed that under 5 mg/L melatonin treatment, chlorophyll content and plant fresh weight increased significantly, while relative conductivity decreased significantly. These changes in physiological parameters suggest that melatonin can enhance photosynthetic efficiency in in vitro cultured tissues at high temperatures, mitigate oxidative damage by scavenging reactive oxygen species, and prevent water loss [26,27,28]. In summary, these results demonstrate that exogenous melatonin treatment significantly boosts the heat tolerance of *R. hybrida* plants and in vitro cultured tissues.

### 3.2. RhCOMT1 Responds to HT and Exogenous Melatonin Through Changes at Both Transcriptional and Post-Transcriptional Level

The heat stress responsiveness of *RhCOMT1* in *R. hybrida* was investigated through exposure to high-temperature conditions, with subsequent analysis of gene expression patterns. Interestingly, *RhCOMT1* expression did not show a linear correlation with the duration of heat treatment. Specifically, treatments lasting 1 h, 6 h, and 12 h led to a decrease in *RhCOMT1* expression in leaves, while treatments lasting 24 h and 48 h resulted in a significant upregulation of expression (Figure 2A). These results suggest that induction of *RhCOMT1* may occur in response to more severe heat stress, consistent with findings from studies on COMT in response to abiotic stresses in other plant species such as soybean [19], rice [18], and tomato [14]. Furthermore, existing evidence indicates that exogenous melatonin treatment can enhance the expression of genes involved in melatonin synthesis, leading to increased endogenous melatonin levels [21,29]. Our study confirmed that treatment with melatonin at a concentration of 5 mg/L upregulated *RhCOMT1* expression in in vitro *R. hybrida* cultures (Figure 2B). Remarkably, plants treated with this concentration exhibited the highest level of heat tolerance. These results suggest that exogenous melatonin can stimulate the expression of key genes related to endogenous melatonin synthesis in vivo, thereby enhancing plant heat tolerance.

The subcellular localization of *RhCOMT1* was investigated, revealing its translocation from the cytoplasm to chloroplasts under heat stress conditions (Figure 3). Previous research has established the presence of COMT in the cytoplasm [30]. Within the melatonin synthesis pathway, SNAT, localized in chloroplasts, catalyzes the conversion of serotonin to NAS, a process requiring COMT for melatonin production [30]. Additionally, the overexpression of rice COMT in chloroplasts has been shown to elevate 5-MT levels and melatonin synthesis [31]. It is postulated that the relocation of COMT to chloroplasts during high-temperature treatment aims to enhance melatonin biosynthesis in these organelles. Given melatonin’s role in enhancing the stability of photosynthetic pigments like chlorophyll, promoting the photosynthetic electron transport chain (PET), and stimulating D1 protein synthesis [32], the localization of *RhCOMT1* suggests a potential protective function within chloroplasts.

### 3.3. Exogenous Melatonin Reduces R. hybrida Oxidative Stress by Activating Melatonin Synthesis Gene RhCOMT1

**Figure 6:**
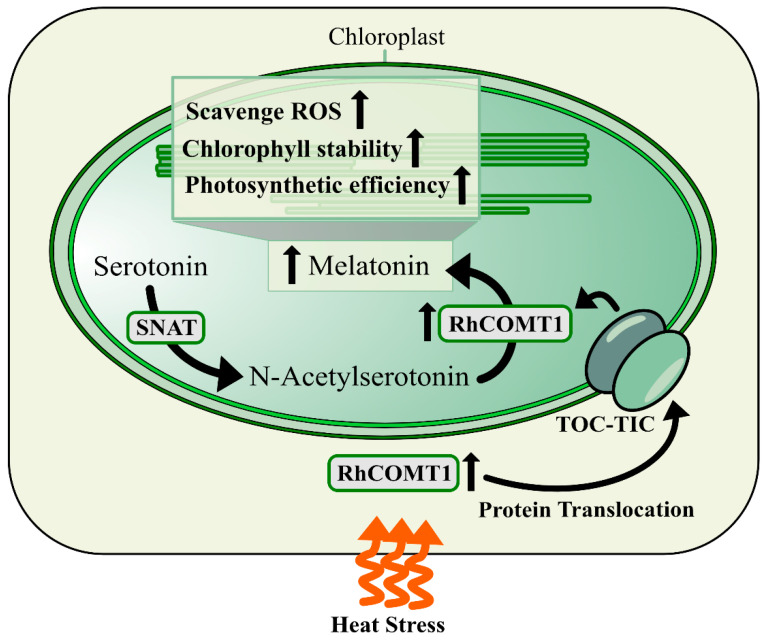
Model for *RhCOMT* responding to heat stress of *R. hybrida*. *RhCOMT1* can be significantly induced by heat stress. The over-expression of RhCOMT1 has been shown to enhance heat stress tolerance by increasing the capability to scavenge ROS. Furthermore, RhCOMT1 has been observed to localise in chloroplasts at high temperatures, which has the potential to improve chloroplast chlorophyll stability and photosynthetic efficiency.

The histochemical staining in this study clearly demonstrated that *RhCOMT1* overexpression in petals resulted in a significant reduction in H_2_O_2_ accumulation compared to the control group (pSuper1300) under heat stress conditions (Figure 4). Conversely, silencing of *RhCOMT1* led to a marked increase in H_2_O_2_ levels in the plants (Figure 5). H_2_O_2_ is a reactive oxygen species (ROS) generated through the reaction of superoxide anions [33]. Given the known association between ROS levels and internal homeostasis, it was speculated that the upregulation of RhCOMT1 may contribute to the enhanced petal health during heat stress. Previous studies have shown that overexpression of SlCOMT1 in tomatoes led to elevated melatonin levels [34] and that exogenous melatonin or COMT overexpression can mitigate melatonin deficiency in tomatoes [35]. In the current investigation, the impact of *RhCOMT1* overexpression and knockdown on oxidative stress appears to be linked to its role in regulating melatonin synthesis. Furthermore, as evidenced by the regulation of *RhCOMT1* expression by exogenous melatonin (Figure 2B), it is plausible that exogenous melatonin may enhance endogenous melatonin levels, reduce oxidative stress, and enhance heat tolerance in *R. hybrida* by activating the expression of genes involved in endogenous melatonin synthesis. We propose a model of how *RhCOMT1* functions in heat stress. Elevated temperatures induce an upregulation in RhCOMT1 expression, leading to its localization in chloroplasts. This, in turn, stimulates chlorogenic acid synthesis, boosting ROS metabolism and enhancing chlorophyll stability. Consequently, photosynthetic efficiency is heightened, thereby promoting overall plant health (Figure 6).

This study focuses on the regulation of rose gene expression in response to exogenous melatonin treatment, identifying a gene, *RhCOMT1*, responsive to exogenous melatonin treatment. The analysis revealed its role in enhancing reactive oxygen species metabolism and promoting plant health under high temperatures. Future research will further investigate the relationship between *RhCOMT1* expression levels and endogenous melatonin content in roses, enriching our understanding of the physiological mechanisms by which melatonin enhances rose heat tolerance. Additionally, studying the expression patterns of *RhCOMT1* under the combined influence of high temperatures and exogenous melatonin will be crucial for unraveling the molecular mechanisms underlying rose responses to heat stress.

## 4. Materials and Methods

### 4.1. Plant Materials and High-Temperature Treatment

One-year-old *R. hybrida* ’Samantha’ seedlings were subjected to high-temperature treatments during flowering period. The seedlings were trimmed and grown under 16 h light/8 h dark photoperiod at 25 °C for 50 d until uniform 9 mm buds were obtained. Prior to bud expansion, the seedlings were divided into three equal portions and placed in an artificial climate chamber for different treatments. One portion served as the control group (CK) and was maintained at 25 °C with distilled water spraying, while the other two portions were sprayed with either 0 or 232 mg/L (100 μmol/L) melatonin at 42 °C high-temperature treatment (HT) until runoff (8 d). Max flower diameter and petal count were recorded at peak bloom in both control and treatment groups.

Sterile tissue-cultured *R. hybrida* ‘Samantha’ plantlets were treated with melatonin concentrations of 0, 1, 2.5, 5, 7.5, and 10 mg/L. The plantlets were cultured on MS medium at 22 °C under a 16 h light/8 h dark photoperiod. After 40 d of growth, plantlets measuring 3–3.5 cm were transferred to various melatonin concentrations (refer to Table S1) for 48 h and then divided into four groups. One group was promptly frozen in liquid nitrogen and stored at −80 °C for subsequent analysis of *RhCOMT1* expression patterns. The remaining groups were exposed to a temperature of 42 °C for 12 d in an artificial climate chamber. Subsequently, their fresh weight, chlorophyll content, and relative ion leakage were evaluated using the method established by Li (2000) [36].

*Nicotiana benthamiana* (tobacco) seeds were planted in plastic pots, germinated, and cultivated in an incubator under conditions of 25 °C and a 16 h light/8 h dark photoperiod.

### 4.2. Cloning of RhCOMT1 from R. hybrida ‘Samantha’ and RT-qPCR

Total RNA was extracted from leaves of one-year-old cutting seedlings of *R. hybrida* ‘Samantha’ exposed to high-temperature treatment at 42 °C for 48 h using the hot borate method [37]. Subsequently, 1 μg of total RNA was utilized to synthesize cDNA employing the HiScript II 1st Strand cDNA Synthesis Kit (Vazyme Biotech, Nanjing, China). A 1182 bp fragment containing the ORF (1062 bp) of *RhCOMT1* was amplified with specific primers listed in Table S2. RT-qPCR analysis was conducted on the StepONE Plus system (Applied Biosystems, Waltham, MA, USA) using FastSYBR Mixture (Cowin Biotech, Taizhou, China) and specific primers (Table S2). Data analysis was performed using the 2^−ΔΔCT^ method [38], with the internal control gene being *RhUBI1*.

### 4.3. Phylogenetic Tree Analysis and Multiple Sequence Alignment

The complete *RhCOMT1* (*RchiOBHmChr7g0187791*) sequence was isolated from *R. hybrida* ‘Samantha’. Amino acid sequences were obtained from NCBI (https://www.ncbi.nlm.nih.gov/) and utilized in MEGA X to construct a phylogenetic tree employing the neighbor-joining method with 1000 bootstrap replicates [39]. Multiple amino acid sequence alignment was conducted using Muscle. Three-dimensional structural analysis and identification of conserved protein domains were performed using the online software SWISS-MODEL (https://swissmodel.expasy.org/).

### 4.4. Subcellular Localization Assays

Subcellular localization assays were conducted by inserting the coding region of *RhCOMT1* into the pSuper1300-GFP vector at the SalI and KpnI sites, resulting in the creation of pSuper1300:GFP-*RhCOMT1* fusion protein using specific primers listed in Table S2. Reconstructed and empty vectors were individually introduced into Agrobacterium tumefaciens GV3101 and infiltrated into 4-week-old *Nicotiana benthamiana* (tobacco) leaves. Following a 24-h dark incubation period, plants expressing *RhCOMT1*-GFP were subjected to two different temperature conditions (25 °C and 42 °C) for 48 h under a 16 h light/8 h dark photoperiod. Subsequently, the lower epidermis of *N. benthamiana* leaves was visualized using a Zeiss LSM 980 confocal laser scanning microscope with a 63× oil immersion lens (Zeiss, Oberkochen, Germany) and Z1/7 imaging system (Thermo Fisher, Waltham, MA, USA). Chloroplasts containing GFP signals were excited within a detection wavelength range of 300 to 700 nm, while GFP itself was excited within a range of 500 to 600 nm.

### 4.5. Silencing of RhCOMT1 in Rose Petals Through VIGS Assay

A 314 bp fragment specific to *RhCOMT1*, comprising 120 bp of 3′UTR and 194 bp of CDS regions, was PCR-amplified and cloned into the expression vector pTRV2 using EcoRI and BamHI restriction sites, resulting in pTRV2-*RhCOMT1*. Subsequently, pTRV1, pTRV2, and pTRV2-*RhCOMT1* were individually introduced into Agrobacterium strain GV3101. The Agrobacterium colonies were cultured overnight in Luria–Bertani medium supplemented with 10 mM MES (pH 6.3), 20 μmol/L acetosyringone, 50 μg/mL kanamycin, and 50 μg/mL gentamycin sulfate. After centrifugation at 4000 rpm for 10 min, the cells were resuspended in infiltration buffer (10 mM MgCl2, 10 mM MES, 200 μmol/L acetosyringone, pH 5.6) to reach a final OD600 of 1.5. A mixture of pTRV1 and pTRV2 cultures in a 1:1 (*v*/*v*) ratio was then incubated in the dark at room temperature for 3 h.

Silencing of *RhCOMT1* in rose petals was achieved through virus-induced gene silencing (VIGS). The outer petals of unopened flowers of rose ‘Bride’ were removed, and 1 cm diameter discs were excised from the inner petals using a hole punch. These discs were immersed in the bacterial solutions and infiltrated under negative pressure (−0.7 MPa, 5 min), followed by washing and incubation in sterile water (96 h, 8 °C, dark). For high-temperature treatment, the discs were exposed to 42 °C for 36 h. The color changes in the petal discs were observed and recorded. The presence of high temperature induced the accumulation of intracellular peroxides, causing the oxidation and degradation of pigments. Materials with lower thermotolerance displayed accelerated color fading under high-temperature stress, highlighting that the decrease in pigments correlates with the thermotolerance level of plants [40]. Subsequently, both treated and untreated petal discs were analyzed for relative expression levels of *RhCOMT1*, H_2_O_2_ content, and relative ion leakage. The measurement of relative ion leakage was conducted following the method established by Li (2000) [36], as outlined in Section 4.1, while the content of H_2_O_2_ was also determined via DAB staining, following the methodology established by Daudi et al. (2012) [41] in earlier research

### 4.6. Transient Overexpression Assay in R. hybrida Petals

For the transient overexpression assay in rose petals, the expression vector pSuper1300:GFP-*RhCOMT1*, previously employed in subcellular localization assays, was utilized. Following this, pSuper1300:GFP and pSuper1300:GFP-*RhCOMT1* were introduced into Agrobacterium strain GV3101. The primer information is provided in Table S2, and additional steps can be referenced in Section 4.5 for coherence.

## 5. Main Conclusions

To summarize, the study indicates that exogenous melatonin enhances the thermal tolerance of *R. hybrida*, likely by modulating *RhCOMT1* expression. The heightened *RhCOMT1* expression during high-temperature stress helps reduce oxidative stress in plants, contributing to overall plant well-being.

## Data Availability

Data are contained within this article.

## Supplementary Materials

The following supporting information can be downloaded at https://www.mdpi.com/article/10.3390/plants14010029/s1. Figure S1: Expression pattern of *RchiOBHm_Chr1g0382961* under high-temperature treatment; Figure S2: Multiple amino acid sequence alignment of COMT proteins; Figure S3: Subcellular localization of RhCOMT under RT and HS; Table S1: Melatonin medium; Table S2: The primer sequences used in this study.
