# Supplementary material for: Exogenous Melatonin Boosts Heat Tolerance in Rosa hybrida via RhCOMT1 Modulation
Source: Plants (Basel). 2024 Dec 25;14(1):29. doi: 10.3390/plants14010029 (PMC11722804; doi:10.3390/plants14010029)
Supplement: Supplementary file 1 [file plants-14-00029-s001.zip › plants-3338336-supplementary.pdf]

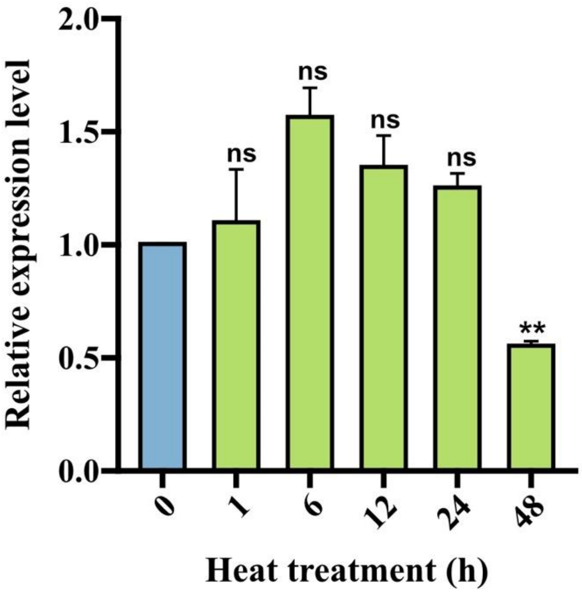

Figure S1. Expression pattern of *RchiOBHm_Chr1g0382961* under high-temperature treatment. The data are presented as the means ± SDs (n = 9). Significant differences compared to the first bar are indicated by asterisks (*P < 0.01, **P < 0.01, ***P < 0.001, student's t-test).

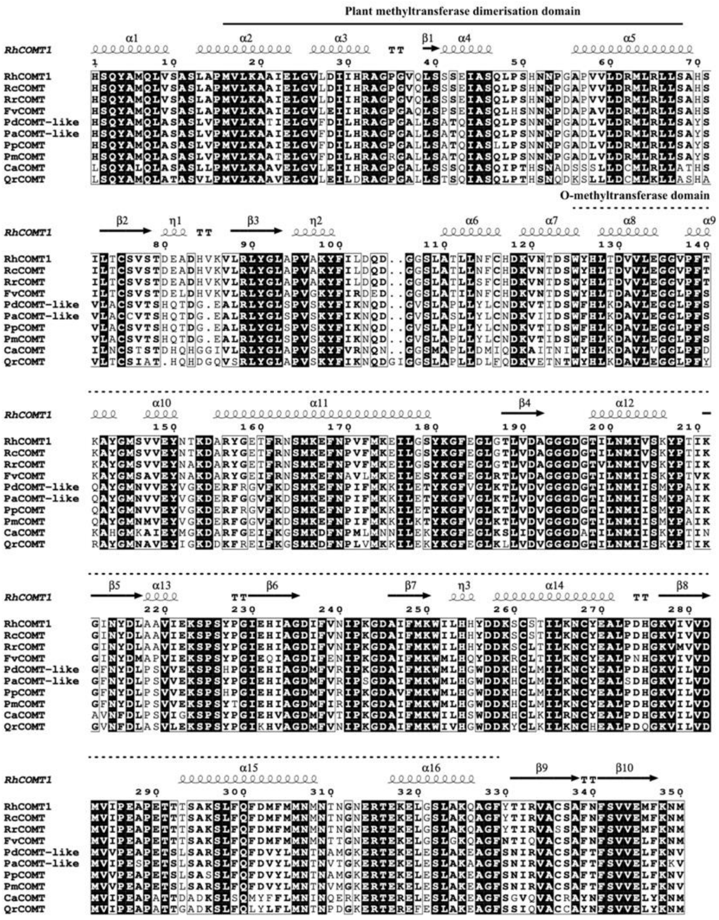

Figure S2. Multiple amino acid sequence alignment of COMT proteins.

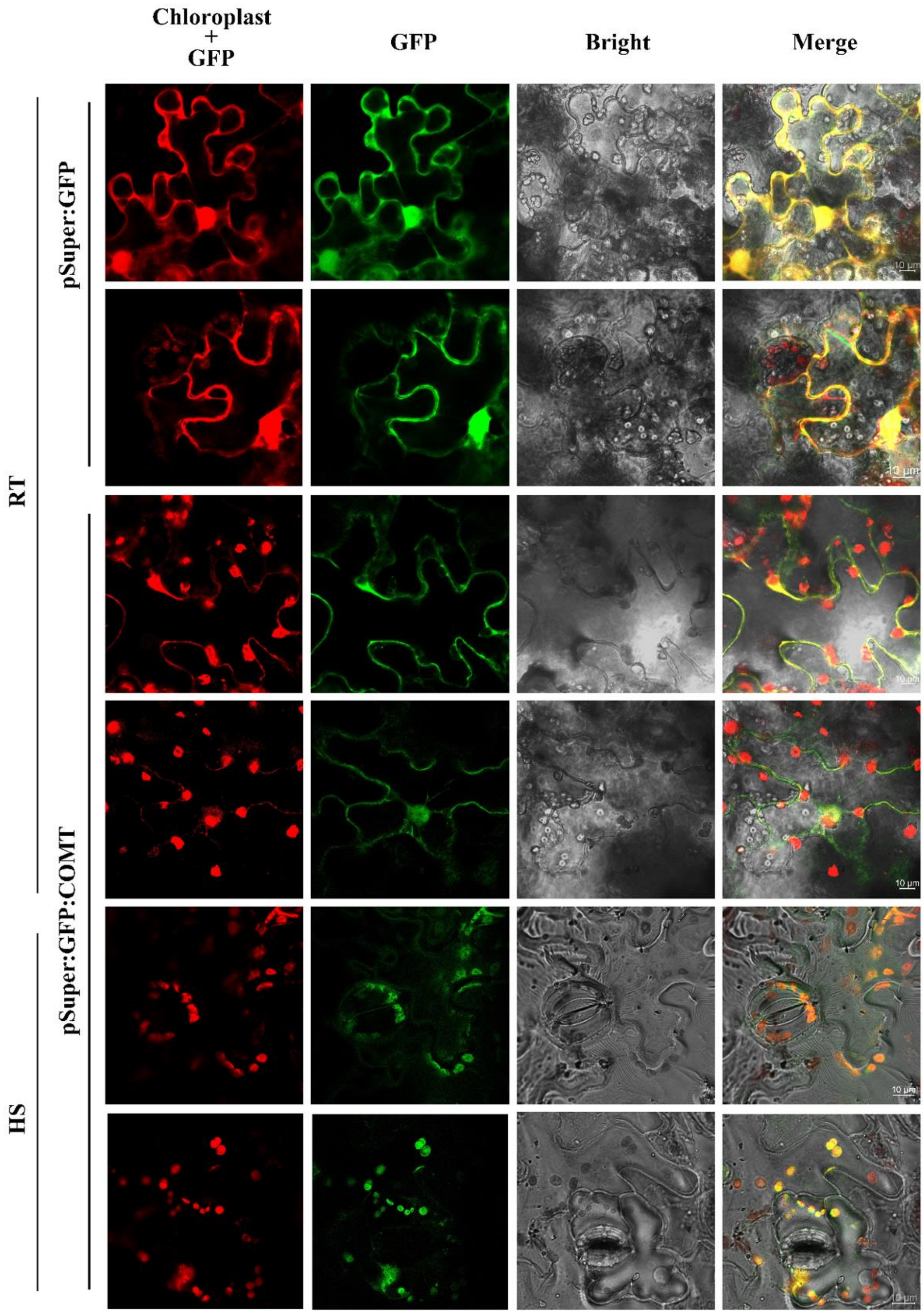

Figure S3. Subcellular localization of RhCOMT under RT and HS. The location of RhCOMT is based on visualization of GFP in tobacco leaves transformed with a fusion construct (pSuper:GFP-RhCOMT) or empty vector (pSuper:GFP). Microscopic images were taken under bright filed and fluorescence. The overlapped images are shown on the right. RT, room temperature ; HS, heat stress ; GFP, green fluorescent protein.

Table S1. Melatonin medium

Ingredient	Quantity
Murashige & Skoog	4.4g/L
Sucrose	30g/L
Melatonin	0, 1, 2.5, 5, 7.5, 10 μmol/L
Agar powder	8.5g/L
pH	5.9

Table S2. The primer sequences used in this study.

Primer name	Sequence(5'-3')
RhCOMT1 cloning primer-1182bp-F	CGTAACCCAGTTGGACACC
RhCOMT1 cloning primer -1182bp-R	TTACATGTTCTTGAACATCTCCACC
RhCOMT1 silencing primer -TRV2-314bp-F	gtgagtaaggttaccgaattcCGTAACCCAGTTGGACACCATC
RhCOMT1 silencing primer -TRV2-314bp-R	cgtgagctcggtaccggatccCGATCAAGCACTACAGGAGCAC
RhCOMT1 overexpression/ subcellular localization primer	gacgagctgtacaaggtcgacATGGACAATCACTCCCAATATGC
RhCOMT1 overexpression/ subcellular localization primer	gtctttgtagtccatggtaccTTACATGTTCTTGAACATCTCCACC
RhCOMT1-qPCR-F	CGTAACCCAGTTGGACACC
RhCOMT1-qPCR-R	CGATCAAGCACTACAGGAGCA
RhUBI1-qPCR-F	GGGCAATCATCTGGAAGTGCTCGT
RhUBI1-qPCR-R	GCCCCCAAAGAGAAACCCTGCG
RchiOBHm_Chr1g0382961-qPCR-F	CACTCCACCAACCCCAAAATG
RchiOBHm_Chr1g0382961-qPCR-R	CTTGGCCATGATCTCCAAG
